# Patient Triage and Guidance in Emergency Departments Using Large Language Models: Multimetric Study

**DOI:** 10.2196/71613

**Published:** 2025-05-15

**Authors:** Chenxu Wang, Fei Wang, Shuhan Li, Qing-wen Ren, Xiaomei Tan, Yaoyu Fu, Di Liu, Guangwu Qian, Yu Cao, Rong Yin, Kang Li

**Affiliations:** 1 West China Biomedical Big Data Center West China Hospital Sichuan University Chengdu China; 2 Department of Industrial Engineering Sichuan University Chengdu China; 3 Department of Nursing West China School of Medicine Sichuan University Chengdu China; 4 Department of Medicine Queen Mary Hospital University of Hong Kong Hong Kong China (Hong Kong); 5 Med-X Center for Informatics Sichuan University Chengdu China; 6 Department of Computer Science Sichuan University Chengdu China; 7 Department of Emergency Medicine West China Hospital of Sichuan University Chengdu China

**Keywords:** ChatGPT, artificial intelligence, patient triage, health care, prompt engineering, large language models, Modified Early Warning Score

## Abstract

**Background:**

Emergency departments (EDs) face significant challenges due to overcrowding, prolonged waiting times, and staff shortages, leading to increased strain on health care systems. Efficient triage systems and accurate departmental guidance are critical for alleviating these pressures. Recent advancements in large language models (LLMs), such as ChatGPT, offer potential solutions for improving patient triage and outpatient department selection in emergency settings.

**Objective:**

The study aimed to assess the accuracy, consistency, and feasibility of GPT-4–based ChatGPT models (GPT-4o and GPT-4-Turbo) for patient triage using the Modified Early Warning Score (MEWS) and evaluate GPT-4o’s ability to provide accurate outpatient department guidance based on simulated patient scenarios.

**Methods:**

A 2-phase experimental study was conducted. In the first phase, 2 ChatGPT models (GPT-4o and GPT-4-Turbo) were evaluated for MEWS-based patient triage accuracy using 1854 simulated patient scenarios. Accuracy and consistency were assessed before and after prompt engineering. In the second phase, GPT-4o was tested for outpatient department selection accuracy using 264 scenarios sourced from the Chinese Medical Case Repository. Each scenario was independently evaluated by GPT-4o thrice. Data analyses included Wilcoxon tests, Kendall correlation coefficients, and logistic regression analyses.

**Results:**

In the first phase, ChatGPT’s triage accuracy, based on MEWS, improved following prompt engineering. Interestingly, GPT-4-Turbo outperformed GPT-4o. GPT-4-Turbo achieved an accuracy of 100% compared to GPT-4o’s accuracy of 96.2%, despite GPT-4o initially showing better performance prior to prompt engineering. This finding suggests that GPT-4-Turbo may be more adaptable to prompt optimization. In the second phase, GPT-4o, with superior performance on emotional responsiveness compared to GPT-4-Turbo, demonstrated an overall guidance accuracy of 92.63% (95% CI 90.34%-94.93%), with the highest accuracy in internal medicine (93.51%, 95% CI 90.85%-96.17%) and the lowest in general surgery (91.46%, 95% CI 86.50%-96.43%).

**Conclusions:**

ChatGPT demonstrated promising capability for supporting patient triage and outpatient guidance in EDs. GPT-4-Turbo showed greater adaptability to prompt engineering, whereas GPT-4o exhibited superior responsiveness and emotional interaction, which are essential for patient-facing tasks. Future studies should explore real-world implementation and address the identified limitations to enhance ChatGPT’s clinical integration.

## Introduction

In China, hospitals generally do not rely on scheduled appointments, and many patients present without prior reservation and register at the registration center upon arrival [1]. This situation is more common in small hospitals or community hospitals. This presents a potential issue. The registration process is primarily self-service, leading to confusion among many individuals when selecting the appropriate department, particularly among the general public with limited health care knowledge. As a result, some patients tend to go to emergency departments (EDs) even though their cases may not be urgent. EDs play a crucial role in the Emergency Medical Service System, delivering acute care for a variety of urgent health issues, such as cerebral hemorrhage, cardiac arrest, and severe injuries. In China, the process of a patient’s visit to the ED typically starts from initial triage at the triage table or reception desk. Based on the health condition, the patient is then directed to either the resuscitation area for urgent care or the general consultation area if the situation is less critical. Around the world, different kinds of triage systems for EDs have been developed to quantitatively measure the priority of patients visiting EDs, and each system may have distinct criteria and focuses [2]. However, the EDs in China do not share a common triage guidance, and the criteria may differ between hospitals [3].

Modified Early Warning Score (MEWS), first proposed in 1999, is a scoring metric based on crucial patient parameters [4]. It is a physiological scoring system used to assess whether a patient’s condition is stable or requires immediate medical intervention. The original application of MEWS in health care was in wards, and physicians could assess if a patient’s health condition is stable enough for transfer. Later applications expanded to the scenarios of intensive care units (ICUs) to identify deteriorating patients [4,5]. EDs are now increasingly using MEWS as a crucial factor in determining the triage process for patients. This scoring system helps medical staff quickly assess the severity of a patient’s health condition and prioritize treatment based on the level of urgency [2,6,7]. In EDs, the majority of physicians in the general consultation area are general practitioners. This arrangement means that patients with less severe cases visiting EDs are not required to select a specific department; instead, they are directly attended to by a general practitioner. While this approach may seem convenient for these patients, it may contribute to overcrowding in EDs. During peak hours in the ED, nurses at the reception desk often advise patients who do not require immediate care to switch to the outpatient area to ease the pressure on the ED. Research conducted in China found that from 2006 to 2018, the number of ED visits increased from 51.9 million to 166.5 million [8]. The rapidly increasing number of visits has led to 4 major challenges: overcrowding, prolonged length of stay, poor work environment, and work exhaustion [8]. Moreover, the shortage of physicians has further exacerbated this situation [9].

Large language models (LLMs) are typically trained on billions of words from, for example, research articles, books, records, and other contents that are internet-based to process natural language and engage in human-like conversation [10]. This process requires deep learning to characterize the inner relationships between words in sentences, and when more text-based training data are provided, the model becomes more accurate and advanced [11]. In recent years, research on LLM applications has expanded significantly, ranging from basic sentence prediction to aiding human decision-making. The potential of LLMs is being increasingly realized with the continuing advancement of artificial intelligence (AI).

First released to the public in November 2022 by OpenAI, ChatGPT represents a significant milestone in making LLMs accessible to the general public. Due to its accuracy and ease of use, applications of ChatGPT in assisting humans have been advantageous. With the ongoing shortage of physicians and growing pressure on public health care resources [12], the implementation of “digital doctors,” which are computer-based chatbots or diagnostic algorithms, seems urgent for governments to alleviate the strain on medical resources. Existing research regarding the use of ChatGPT in the medical field has demonstrated high performance and reliability in specific application scenarios [13]. Additionally, the improvement is significant when shifting from GPT-3.5 (released in 2022) to GPT-4 (released in 2023). In taking the United States Medical Licensing Exam (USMLE) Step 2 Clinical Knowledge (CK), GPT-4–based ChatGPT had an overall accuracy of 87.4%, while GPT-3.5–based ChatGPT had an accuracy of only 47.7% [14]. In addition to providing support in English-speaking countries, GPT-4 can function as a medical assistant in non-English–speaking countries [15]. Therefore, employing LLMs like ChatGPT in the medical field is promising. GPT-4o was first introduced on May 13, 2024, and it has been reported by OpenAI to have swifter responses and improved performance compared to previous models [16]. GPT-4-Turbo (released in 2023) and GPT-4o are 2 different variations of GPT-4. However, the actual performance of GPT-4o compared to GPT-4-Turbo has not yet been thoroughly verified, leaving some uncertainty about the relative strengths and capabilities of these models.

To address ED challenges, the integration of AI in assisting medical decisions may help reduce the workload of physicians and minimize the potential for medical errors associated with extended working hours. Previous research suggested that GPT-3.5–based ChatGPT showed relatively poor performance and low consistency in patient triage using the Canadian Triage and Acuity Scale [17]. However, there is limited research regarding the application of GPT-4–based ChatGPT in helping triage and providing guidance.

Our research aims to fill the research gap in using GPT-4–based ChatGPT for assisting with triage in EDs. Simultaneously, it seeks to provide guidance on selecting appropriate departments in the outpatient area if necessary. This strategy is intended to provide suggestions for redirecting patients with minor symptoms to outpatient services, thereby alleviating the heavy pressure on EDs. A 2-phase experiment was conducted to evaluate the capabilities of ChatGPT in providing comprehensive assistance at the reception of EDs. Initially, the accuracy and consistency of ChatGPT’s triage abilities based on MEWS were assessed. In the second phase, the focus shifted to evaluating the accuracy and consistency of ChatGPT’s ability to provide advice to patients with less urgent health issues regarding the selection of appropriate outpatient departments.

## Methods

### Patient Triage Evaluation

Our research adopts MEWS, one of the most commonly used metrics for triage criteria in mainland China [3,18]. MEWS uses 4 distinct physiological measurements and 1 subjective measurement to reflect the patient’s current condition. These include systolic blood pressure (in mmHg), heart rate (in beats per minute), respiratory rate (in breaths per minute), body temperature (in °C), and AVPU (alert, response to voice, response to pain, unresponsive) score, which assesses the patient’s level of consciousness [19,20]. For each parameter, the readings are categorized into 4 tiers (0-3), with each tier representing a specific score for that parameter. These tiers help quantify the severity of a patient’s condition. The chatbot should sum up the scores of all parameters to obtain the final score, and the triage evaluation process is to ensure that the chatbot can perform triage and department selection guidance at the same time. Detailed information is provided in Table 1 [19].

Data were taken from previous MEWS research regarding patient MEWS scores in Vrije Universiteit University Medical Center [20]. The dataset includes a total of 3673 data entries. For each entry, detailed scores for each parameter and the total MEWS score are provided. After excluding entries with incomplete data that lacked sufficient critical parameters to calculate the MEWS score, the final dataset for the triage evaluation consisted of 1854 entries. The refined dataset ensured a more accurate and reliable analysis of triage evaluation based on the MEWS grading in Table 1. A total of 1854 patients were simulated, with detailed values for each parameter described in the MEWS derived directly from the dataset. Preliminary experiments were conducted using the following unrevised prompt in Chinese: “From now on, you should act as a nurse in the reception area of the Emergency Department of a Hospital. You should use the Modified Early Warning Score (MEWS) scoring metric to evaluate each patient. For each patient, the critical parameters are listed as follows. You should provide a MEWS score for each patient.” This prompt was designed to simulate the role of a nurse using MEWS to assess the urgency of care required by each patient based on specified critical parameters. It is important to note that all experiments in the triage evaluation were conducted using 2 specific variations of GPT-4: GPT-4o and GPT-4-Turbo. The performances of GPT-4o and GPT-4-Turbo were compared and analyzed. To ensure a fair comparison, the same prompts and datasets were provided for each model. Subsequently, the differences in consistency, accuracy, and overall performance between the 2 versions under identical testing conditions were assessed.

**Table 1:** Grading of Modified Early Warning Score [19].

Parameter	Tier (score)^a^						
	3	2	1	0	1	2	3
Systolic blood pressure (mmHg)	≤70	71-80	81-100	101-199	—^b^	≥200	—
Heart rate (beats per min)	—	≤40	41-50	51-100	101-110	111-129	≥130
Respiratory rate (breaths per min)	—	≤8	—	9-14	15-20	21-29	≥30
Temperature (°C)	—	≤34	—	35-38.4	—	≥38.5	—
AVPU^c^ score	—	—	—	Alert	Reacting to voice	Reacting to pain	Unresponsive

^a^Detailed scoring thresholds for key physiological parameters used in the Modified Early Warning Score assessment. A patient’s total score is the sum of the scores of all parameters.

^b^Not applicable.

^c^AVPU: alert, response to voice, response to pain, unresponsive.

**Figure 1:**
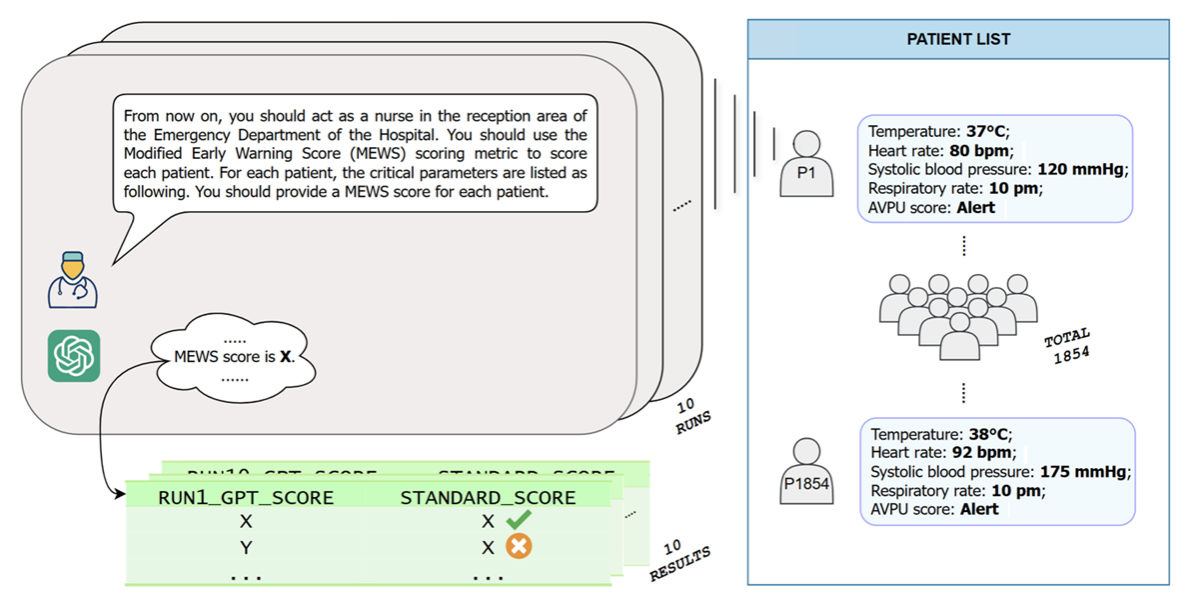
Illustration of the preliminary study design. This figure outlines the study setup for scoring patients using the Modified Early Warning Score (MEWS) in a simulated hospital emergency department environment. ChatGPT was instructed to act as a nurse, tasked with calculating MEWS scores based on given patient parameters. The system is instructed to conduct 10 runs, each generating a MEWS score for each patient. Patient data include key parameters necessary for MEWS calculation: temperature, heart rate, systolic blood pressure, respiratory rate, and AVPU score, which assesses awareness levels. A total of 1854 patient scenarios have been included in the study. The resulting MEWS scores are recorded, allowing for comparison between generated and standard scores across multiple runs. AVPU: alert, response to voice, response to pain, unresponsive.

In the preliminary test, each simulated patient was inputted into GPT-4 (both GPT-4o and GPT-4-Turbo) 10 times, resulting in a total of 18,540 pieces of data collected for each model. The outcomes were later compared with standard scores to evaluate accuracy. In this study, “standard score” refers to the patient’s correct MEWS score, which is the established, verified score calculated using standard criteria. This score serves as a reference for comparing ChatGPT-generated outputs. The procedure is shown in Figure 1. This test aimed to assess whether GPT-4–based ChatGPT could perform triage tasks, with a focus on feasibility. Consequently, a few techniques of prompt engineering, which were aimed at improving the model performance by optimizing the prompts provided to the model, were intentionally used, and the prompt provided in the experiment was relevantly ambiguous.

After completing the preliminary experiment, the results were analyzed in 2 dimensions. First, each group of results was compared with the standard score to check the feasibility and accuracy of GPT-4–based ChatGPT in the experiment. The purpose of the comparison was to determine how closely the generated scores matched the established scores. Second, to quantify the consistency between multiple runs of model-generated scores, we employed Kendall correlation coefficients for several reasons [21]. The scoring outcomes represent ordinal rankings rather than continuous measurements. Kendall τ is specifically designed to assess concordance in ranked data, making it particularly appropriate for this application. It effectively measures how consistently the model generates similar scores under identical conditions. After completing the data analysis, the problems and issues identified in the preliminary test were documented. This step is crucial for refining the experimental setup and enhancing the model’s performance in subsequent trials. By addressing these identified problems, improvements could be made to both the accuracy and consistency of the model’s responses.

With the issues collected in the preliminary experiment, prompt engineering was conducted based on the original prompt. Prompt engineering is a method designed to optimize the performance of LLMs, especially on tasks regarding natural language processing [22]. Given the application of ChatGPT in EDs in our study, the major objectives of prompt engineering were high accuracy and high consistency. In the high-stakes environment of EDs, where distinct decisions could significantly affect patient outcomes, even minor errors may lead to unpreventable medical disasters. After the prompt engineering was completed, the revised prompt was given to ChatGPT. Using the same dataset and procedures as those in the preliminary experiment, 18,540 additional responses per model were collected (37,080 responses in total). The second batch of data enabled a comprehensive analysis, including both preliminary and subsequent results. A transversal performance analysis was conducted between GPT-4o and GPT-4-Turbo to assess the reliability, accuracy, and consistency of the models in simulating triage tasks within a controlled experimental setting.

Sample patient triage analysis prompts are presented in Multimedia Appendix 1.

### Department Selection Guidance

The second phase of our research focused on assessing ChatGPT’s accuracy in providing patients with detailed guidance for outpatient department selection. To simulate real-life patient symptoms, the data resource for this phase of the experiment came from a web portal database: Chinese Medical Case Repository [23]. Chinese Medical Case Repository is an open-access repository that includes reports of patient cases and their treatments [23]. Reports containing detailed descriptions of patients’ chief complaints upon admission and the corresponding departments were carefully examined. Physicians extracted the necessary information from these reports, thereby ensuring the accuracy and relevance of the data for our analysis. For each scenario, raw reports were modified to ensure that the patient descriptions matched what would typically be provided at the time of registration. These modifications included medical history, current discomfort, previously received medical care, and basic information. A total of 264 patient scenarios were constructed, covering 33 different kinds of the most common departments, which can be further categorized into 3 major groups (internal medicine department, general surgery department, and other specialized department) in Chinese hospitals, as shown in Textbox 1. Department categorization in this study was based on the standard organizational structure typically found in medium- to large-scale hospitals. Specifically, the classification reflects the common clinical and administrative departments that are universally recognized in such institutions, ensuring that our grouping aligns with conventional hospital management practices. In this part of the experiment, the GPT-4o model was chosen as our primary tool for analysis and testing. GPT-4-Turbo was not considered for this experiment because OpenAI reports that GPT-4o offers faster response speed, offers better foreign language processing ability, and generates more emotional responsiveness during interactions [16]. These factors made GPT-4o more suitable for this user-based task since Chinese was used during the whole interaction process. The prompt provided to GPT-4o–based ChatGPT was as follows (original was in Chinese): “From now on, you are to take on the role of a nurse at the hospital’s registration area, guiding patients based on the description I provide for each case. Your task is to recommend a specific department for the patient's registration. Be mindful that the hospital you work at is a top-tier Chinese hospital with a comprehensive range of departments, including Endocrinology, Digestive System, Neurology, Rehabilitation, Pediatrics, Rheumatology and Immunology, Psychiatry, Cardiology, Urology, Digestive Surgery, Hepatobiliary Surgery, Liver Surgery, Thoracic Surgery, Cardiovascular Medicine, Respiratory Medicine, Critical Care Medicine, Radiology, Oral and Maxillofacial Surgery, Ophthalmology, Otolaryngology, Neurosurgery, Obstetrics, Gynecology, Breast Surgery, Vascular Surgery, Dermatology, Oncology, and Infectious Disease. You need to recommend one specific department from this list based on my description of the patient's symptoms.” For each patient scenario, we tasked ChatGPT with providing specific department selection guidance. To evaluate consistency, each scenario was independently presented to ChatGPT thrice, and for each inquiry, the chatbot did not have the memory of the previous interaction. The response from each run was recorded, along with the word count of each patient’s description, to verify if the length of the patient health description is associated with the accuracy of department selection guidance by ChatGPT. Ultimately, a total of 792 data points were collected. To minimize errors caused by potential memory lapses in ChatGPT, a guidance prompt was provided for each query. This approach ensures that regardless of the length of the chat history or session duration, each interaction with ChatGPT starts with clear instructions, reducing the likelihood of inconsistencies or errors due to the model forgetting previous commands. Moreover, the general performance of GPT-4o during the interaction was subjectively examined by assessing guidance accuracy.

The early symptoms of many diseases are similar, allowing patients to select from various departments for a preliminary check. For instance, patients only reporting diarrhea may visit either the gastroenterology department or digestive disease department. Based on this, the correctness of ChatGPT’s selection guidance for each patient scenario was evaluated and confirmed by 2 physicians. If there was disagreement, a third physician was invited to evaluate the selection guidance. To quantify the responses, correct selection guidance was labeled as “1” and incorrect guidance was labeled as “0.” In this part, logistic regression was conducted to quantitatively address the factors that may have influenced the correctness of ChatGPT’s guidance.

**Departments included for the guidance experiment.**

**Internal medicine department**

- Department of Endocrinology
- Department of Cardiology
- Department of Digestive System
- Respiratory Medicine
- Department of Neurology
- Department of Cardiovascular Medicine
- Department of Rheumatology and Immunology
- Department of Hematology

**General surgery department**

- Department of Urology
- Department of Orthopedics
- Department of Hepatobiliary Surgery
- Department of Respiratory Surgery
- Department of Liver Surgery
- Department of Neurosurgery
- Department of Thoracic Surgery
- Department of Hepato-Pancreato-Biliary Surgery
- Department of Vascular Surgery
- Department of Breast Surgery
- Department of Digestive Surgery

**Other specialized department**

- Department of Rehabilitation
- Department of Gynecology
- Department of Oncology
- Department of Pediatrics
- Department of Critical Care Medicine
- Department of Radiology
- Department of Dermatology
- Department of Psychiatry
- Department of Infectious Disease
- Department of Obstetrics
- Department of Ophthalmology
- Department of Oral and Maxillofacial Surgery
- Department of Otolaryngology

### Data Analysis

The data analysis software used in our research was R (Version 4.3.3; R Foundation for Statistical Computing). The Shapiro-Wilk test was applied to confirm the normality of the datasets, and Wilcoxon rank sum tests were used to compare differences between the model outputs and established baseline scores. Kendall correlation coefficients were examined to assess the consistency of different responses from ChatGPT. The consistency of department selection guidance provided by the GPT-4o model was evaluated using the McNemar test. This test was applied to compare the consistency of ChatGPT’s selection guidance across different runs for the same patient scenarios. Additionally, to further assess the performance improvements between trials, a *t* test was applied to compare the Kendall correlation coefficients from the different trials. A logistic regression model was employed to explore potential correlations between word count in patient descriptions and the correctness of the department selection guidance. This approach allowed for a more nuanced understanding of whether the length of a patient’s symptom description influenced the model’s ability to provide accurate guidance. All queries were executed between April 2024 and May 2024, and all data analyses were conducted between May 2024 and November 2024. A statistical significance level of α=.05 was used.

### Ethical Considerations

This study is based solely on data extracted from previous literature and public databases and does not involve direct interaction with human subjects or primary data collection. All data used in this research were anonymized or deidentified prior to analysis, ensuring that individual patient information could not be traced back to any source, and no personal or sensitive data have been included in our analyses or presented in any images or supplementary materials. As the study did not involve recruiting or interacting with participants, no compensation was provided, and no images or supplementary materials in the manuscript contain the identifiable information of any individual.

## Results

### Automatic Triage Scoring

**Figure 2:**
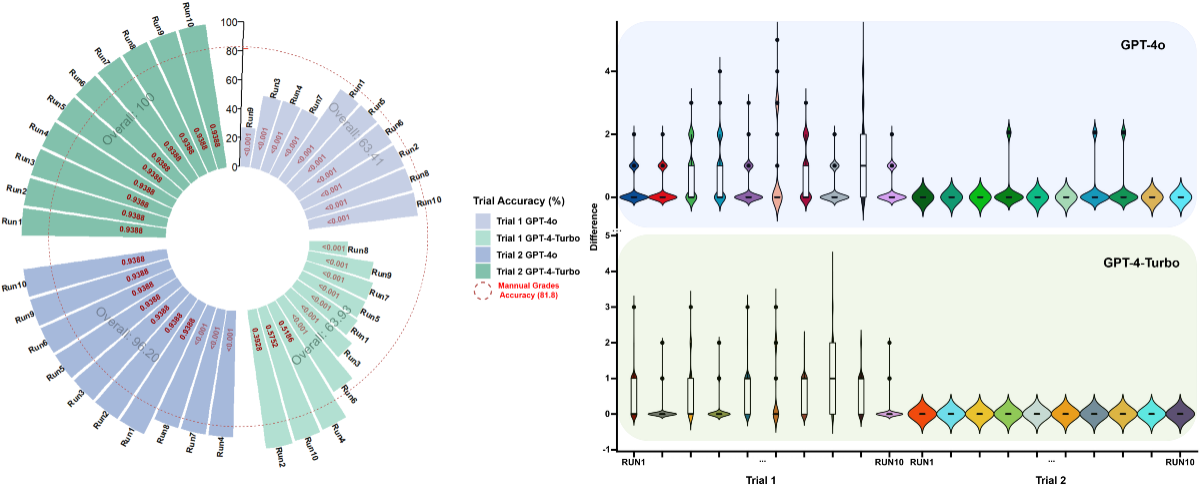
Plots for accuracy of the ChatGPT score and difference between the GPT score and standard score. (A) The circular plot shows the accuracy percentage for each run. Each section represents 10 runs, with accuracy percentages displayed to indicate the consistency of each model’s performance. Manual grade accuracy, represented by a red dashed circle, was set at 81.8%, serving as a benchmark for comparison with GPT-based scoring. (B) Violin plots visualize the distribution of differences between GPT-generated scores and standard scores across each run. Each run’s distribution is represented by a unique color, highlighting the variance in score differences between trials. The width of the box plot indicates the density of score differences for each run, with greater width representing higher density at that specific score difference. The length represents the range of score differences, with a longer length indicating a wider range of differences. The vertical axis displays the difference magnitude, with zero indicating no difference from the standard score. The plots show both trials for each model, revealing differences in the consistency of scoring accuracy between GPT-4o and GPT-4-Turbo models.

For the MEWS-based triage accuracy evaluation, the trials were divided into 2 parts to facilitate data analysis. The first trial (trial 1) included testing 2 models of ChatGPT before applying prompt engineering. The second trial (trial 2) involved testing the 2 models after prompt engineering. In the preliminary experiment (trial 1), the accuracy of each run was measured by dividing the total number of correct scores by the total number of simulated patients. The results are shown in Figure 2. Moreover, Shapiro-Wilk normality tests [24] were conducted for all runs. Our results suggested that the data were not normally distributed (*P*<.001 for all runs). Wilcoxon rank sum tests were conducted between each run result and the standard score group [25], as indicated by red text in Figure 2 for each run. All runs in the GPT-4o group showed a statistically significant difference compared to the standard score (*P*<.001), suggesting the limited performance of ChatGPT-4o without proper prompt engineering. However, 3 out of 10 runs in the GPT-4-Turbo group had no significant difference (*P*=.39, .52, and .58 for run 2, run 4, and run 10, respectively). For GPT-4o, grading accuracy ranged from 27.18% (run 9) to 76.05% (runs 2 and 10), with an overall accuracy of 63.41%. For GPT-4-Turbo, this range was from 26.59% (run 8) to 96.60% (run 2), with an overall accuracy of 63.93%. To present the accuracy of scoring by ChatGPT, the difference between the ChatGPT score and standard score was calculated, using the absolute value of the ChatGPT score minus the standard score for each test group. In Figure 2, a violin plot is used to display the distribution of score differences for each run [26]. The shape of the violin in the graph represents the density of data points at different values. The upper and lower extremes of the violin indicate the maximum and minimum differences observed in each run, respectively. The black vertical line in each violin represents the medium for that dataset, with the upper and lower borders in the white box representing the upper and lower quartiles in the dataset. During trial 1, as illustrated in Figure 2 and Table 2, both GPT-4o and GPT-4-Turbo exhibited scattered performances. GPT-4o generally exhibited a more concentrated clustering of data points around zero difference (*P*<.001 for all runs). This indicates that GPT-4o more frequently matched the standard scores compared to GPT-4-Turbo in trial 1. It is evident that the overall performance of both GPT-4o and GPT-4-Turbo was suboptimal in trial 1, particularly given the application scenario in EDs, which has a relatively high requirement of accuracy. From trial 1, several key areas for improvement were identified. First, the MEWS grading metric used by ChatGPT varied significantly across runs, indicating that the MEWS metric was neither constantly nor accurately used. The detailed MEWS metric should be provided to and clarified for ChatGPT. Second, in several cases, ChatGPT occasionally miscalculated the overall MEWS score. Despite accurately determining individual parameter scores, ChatGPT frequently failed to sum them accurately. Therefore, ChatGPT should be instructed to use some techniques, such as its Python plugin, for mathematical computations. Third, instances were observed where ChatGPT confused parameter readings, such as using the heart rate value for blood pressure. We hypothesized that providing explicit instructions on data usage may be helpful to address this issue. Finally, there were instances where ChatGPT failed to convert the AVPU description into numerical gradings. To address this, the dataset was modified to represent AVPU scores directly as letters, and descriptive words were replaced using the first letter. For instance, reacting to voice was replaced with the single letter V. Detailed presentation of scores and differences can be found in Multimedia Appendix 2.

Addressing the key issues identified in trial 1, a new prompt was developed for trial 2. The results from both GPT-4o and GPT-4-Turbo models in trial 2 showed significant improvement (Figure 2). GPT-4-Turbo achieved an impressive accuracy of 100% across all the 10 runs. The performance of GPT-4o also showed significant improvement in trial 2. Of the 10 runs, 7 achieved 100% accuracy and 3 (runs 4, 7, and 8) achieved an accuracy of 87.32%, with an overall accuracy of 96.20%, which is considered acceptable. Shapiro-Wilk normality tests indicated that the data were not normally distributed (*P*<.001 for all runs). Results from Wilcoxon rank sum tests indicated that after prompt engineering, the difference between the GPT-4-Turbo group and standard score group was no longer significant (*P*=.94 for all runs). For GPT-4o, 3 runs still showed significant differences, while the remaining runs mirrored the results of GPT-4-Turbo (*P*<.001 for runs 4, 7, and 8; *P*=.94 for the other runs).

**Table 2:** Performance of GPT-4o and GPT-4-Turbo on Modified Early Warning Score–based patient triage accuracy before and after prompt engineering.

Variable	Before prompt engineering		After prompt engineering	
	GPT-4-Turbo	GPT-4o	GPT-4-Turbo	GPT-4o
Accuracy^a^	63.93%	63.41%	100%	96.20%
Average difference^b^	0.45	0.54	0	0.08

^a^Manual grade accuracy was set at 81.80%.

^b^Average difference stands for the average value of the difference between the GPT score and standard score.

**Figure 3:**
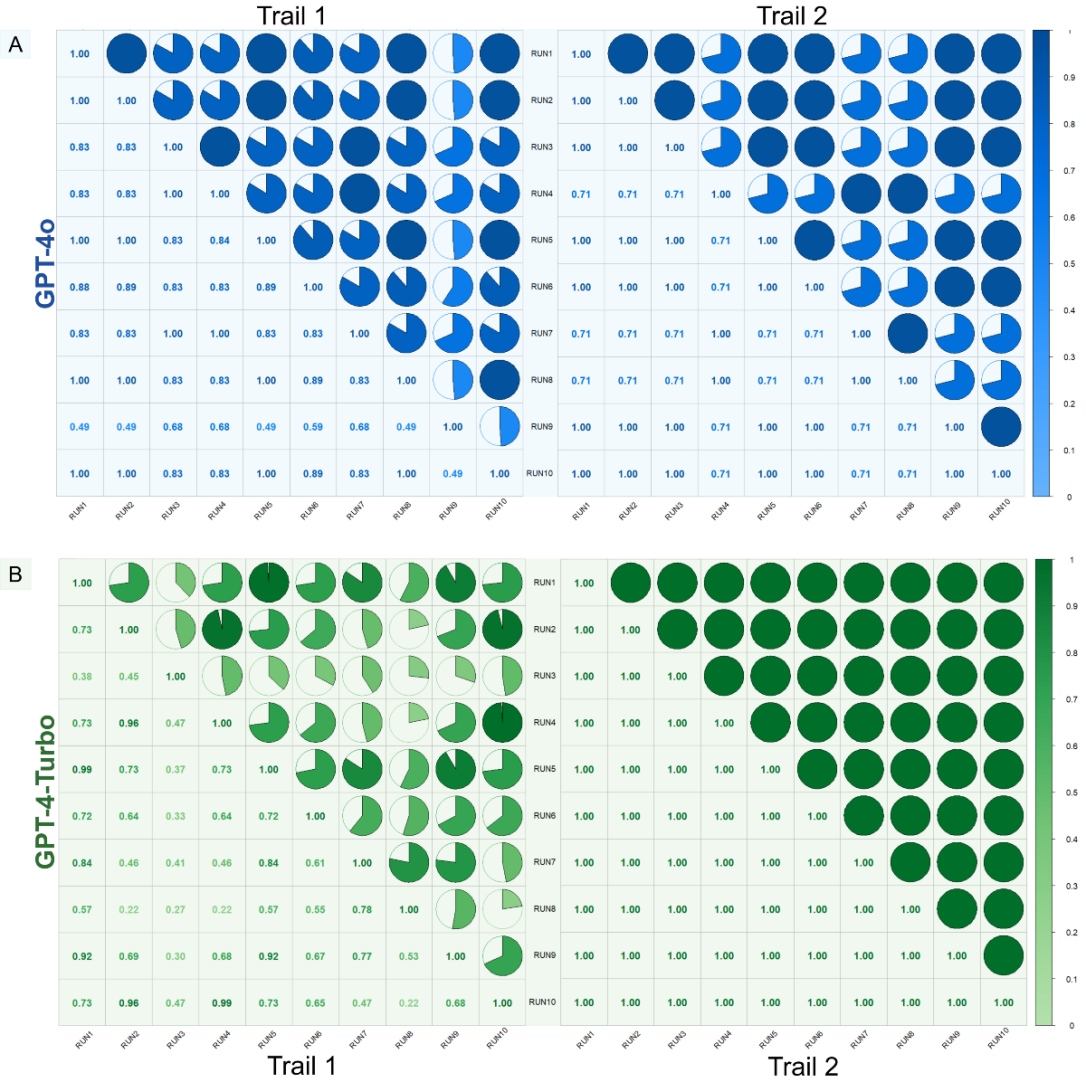
Kendall correlation coefficients between different runs for (A) GPT-4o and (B) GPT-4-Turbo models. Each cell represents the Kendall correlation coefficient between 2 specific runs, with values ranging from 0 to 1, where 1 indicates perfect positive correlation. The color intensity and size of the pie charts within each cell correspond to the strength of the correlation, with darker and fuller circles indicating higher correlation.

To quantitatively measure the consistency in scoring, Kendall correlation coefficients were calculated for both trial 1 and trial 2, and graphed along with detailed results in Figure 3. For instance, after conducting trial 1 for GPT-4o, the consistency among the results of all 10 runs generated by GPT-4o was evaluated. The same procedure was conducted for all trials and models. For GPT-4o, the correlation coefficients in trial 1 ranged from 0.49 to 1, with a mean value of 0.8327 (SD 0.1560). In trial 2, after optimization through prompt engineering, the mean value increased to 0.8646 (SD 0.1447). For GPT-4-Turbo, the mean value dramatically improved from 0.6176 (SD 0.2135) in trial 1 to 1.00 (SD 0) in trial 2. The improvement in the average Kendall correlation coefficients for GPT-4o after optimization was not statistically significant (t_43_=–0.9976; *P*=.32); however, the improvement in GPT-4-Turbo was significant (t_43_=–11.8815; *P*<.001).

### Sample Size Justification

Using the single proportion sample size calculation formula designed by Heinisch and Cochran [27], a sample size of 139 participants was required to estimate the expected proportion with 95% confidence (2-sided test, *Z*=1.96), a margin of error (E) of 0.05, and an anticipated proportion (p) of 0.9, and the formula is as follows:

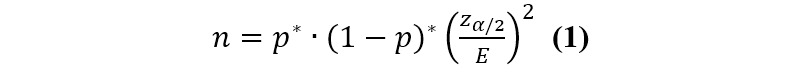



Rounding up to the next whole number, the final sample size was 139. This would ensure sufficient precision to estimate the proportion within ±5% error at a 95% confidence level. The anticipated proportion of 0.9 was selected based on prior assumptions or preliminary data relevant to the study context. In addition, considering a possible sample dropout rate of 20% to 25%, the minimum sample size was set at 167-174. Therefore, our sample size of 264 can be considered sufficient.

### Outpatient Department Selection

The accuracy of department selection guidance provided by GPT-4o was initially assessed. The results for the 3 general department categories are plotted in Multimedia Appendix 3. The overall accuracy for the 3 major departments was 92.63% (95% CI 90.34%-94.93%), with internal medicine departments achieving the highest accuracy of 93.51% (95% CI 90.85%-96.17%). The lowest accuracy rate (91.46%, 95% CI 86.50%-96.43%) was observed in general surgery departments. Performance in other specialized departments was moderate, with an accuracy of 93.18% (95% CI 89.68%-96.68%). Moreover, the consistency between different runs within each department was evaluated using the Wilcoxon test, with detailed results shown in Multimedia Appendix 3. The results indicated high overall consistency, and all comparisons between different runs yielded nonsignificant *P* values (*P*>.1 for all groups), suggesting statistically nonsignificant differences.

For specific departments within each major category, the guidance accuracies for some sample departments are shown in Table 3. The inclusion criteria for this table involved departments with a patient scenario number count greater than 29. Each major category included 3 specific departments that met the inclusion criteria. Among them, the highest accuracy was observed in the Department of Ophthalmology, with an outstanding accuracy rate of 100%. The Department of Dermatology showed the lowest performance overall, achieving an accuracy rate of only 79.57%.

**Table 3:** Specific department selection accuracy of ChatGPT using the GPT-4o model.

Category and specific department			Run 1 accuracy (%)		Run 2 accuracy (%)		Run 3 accuracy (%)		Overall accuracy (%), value (95% CI)
**Internal medicine department**									
	Department of Endocrinology	91.67		91.67		91.67		91.67 (91.67-91.67)	
	Department of Digestive System	90.00		97.50		92.50		93.33 (83.85-100.00)	
	Department of Neurology	95.00		97.50		100.00		97.50 (91.29-100.00)	
**General surgery department**									
	Department of Gastrointestinal Surgery	82.86		91.43		88.57		87.62 (76.78-98.46)	
	Department of Thoracic Surgery	80.00		86.67		83.33		83.33 (75.05-91.61)	
	Department of Orthopedics	83.87		83.87		87.10		84.95 (80.32-89.57)	
**Other specialized department**									
	Department of Ophthalmology	100.00		100.00		100.00		100.00 (100.00-100.00)	
	Department of Dermatology	80.65		74.19		83.87		79.57 (67.32-91.82)	
	Department of Psychiatry	91.89		89.19		89.19		90.09 (86.22-93.96)	

## Discussion

### Result Summary

Our research assessed the performance of ChatGPT (GPT-4o and GPT-4-Turbo models) in providing triage assistance in Chinese EDs using MEWS scoring and analyzed the accuracy of GPT-4o–based ChatGPT in providing guidance on outpatient department selection. The results indicated that the optimized performance of ChatGPT, particularly with the GPT-4o model, was acceptable in both sections of the study. This suggests that ChatGPT is promising for enhancing the overall procedures of ED triage and guidance. The findings support the potential of implementing generative AI to enhance the decision-making processes in EDs, thus improving efficiency and potentially reducing the workload on medical staff by offering triage and departmental guidance.

### Model Selection

In the triage evaluation, GPT-3.5 was initially considered; however, it was ultimately dismissed due to several critical and unsolvable problems during the preliminary experiments conducted with GPT-3.5–based ChatGPT. For example, initially, GPT-3.5 struggled to provide accurate and consistent MEWS scoring. Despite being provided with detailed MEWS metrics, GPT-3.5 was unable to consistently adhere to these guidelines or provide accurate scores. Furthermore, GPT-3.5 exhibited limited mathematical abilities [28]. Even when providing correct scores for individual parameters, GPT-3.5 sometimes failed to accurately sum these scores up to obtain the final MEWS score. Additionally, because Python plugins were not available in GPT-3.5 during our experiments, it could not directly use Python functions for precise numerical calculations. Consequently, significant deviation from the accurate score can occur, which is risky in ED triage applications. Minor deviation of 1 or 2 scores in the MEWS can lead to overlooking patients needing immediate care or providing unnecessary treatment, thus wasting medical resources [29]. Since introducing the GPT-4o model, OpenAI has gradually begun offering public access to GPT-4 for free. Although the current complimentary usage cap is not very high, the availability of the GPT-4 model is expected to continue to expand. This increasing accessibility promises to broaden the application of advanced AI across various fields such as health care. Taking all factors into account, it was preferred to use the more advanced GPT-4 model in our experiment, rather than GPT-3.5.

### Findings of Patient Triage

During the triage evaluation experiment, several interesting findings were noticed. In trial 1, GPT-4o demonstrated superior performance compared to GPT-4-Turbo in terms of both accuracy and consistency. Although the overall accuracies of GPT-4o and GPT-4-Turbo were comparable, GPT-4o demonstrated greater consistency in its responses. The GPT-4-Turbo model exhibited varied MEWS scoring criteria across almost all runs, indicating inconsistency in its responses. In contrast, several runs of GPT-4o–based ChatGPT adhered to consistent MEWS scoring criteria, regardless of the correctness of the criteria it followed. The responses from GPT-4o were more accurate and consistent than those from GPT-4-Turbo.

However, following proper prompt engineering, interestingly, GPT-4o’s performance appeared to be worse than that of GPT-4-Turbo. This was evident as GPT-4-Turbo achieved perfect scores in all 10 runs after same prompt engineering, involving 18,540 queries in total. GPT-4o failed to achieve a 100% accuracy rate in 3 out of 10 runs, although OpenAI has claimed that GPT-4o is the most advanced GPT-4 model currently available [16]. However, our experiment showed that despite its superior performance in trial 1, GPT-4o did not consistently outperform GPT-4-Turbo in trial 2. This suggests that although GPT-4o has significant potential, GPT-4-Turbo may provide more reliable performance under certain conditions or with specific prompt engineering techniques. It is also hypothesized that GPT-4o, being more constrained and finely tuned, exhibited poorer performance than GPT-4-Turbo in areas requiring improvement, suggesting that the additional tuning of GPT-4o may have restricted its flexibility, thereby impacting its ability to adapt and perform optimally under enhanced prompt engineering. Future studies may examine the potential causes of similar issues. These are just preliminary conjectures; future studies should assess the performance of GPT-4o in other applications to further validate these findings. This study also provided a general exploration of applying other LLMs in health care. Generally, in our study, the performance of ChatGPT (GPT-4–based model) in the triage experiment was considered good after prompt engineering. Nevertheless, some minor deviations were observed in GPT-4o even after the enhancement process. According to previous studies, MEWS scores are effective predictors of adverse outcomes and help in critical illness detection, and higher MEWS scores (>3) are significantly related to the occurrence of adverse outcomes [19,20]. Although no universally accepted threshold exists for MEWS scores to definitively indicate whether a patient’s condition is stable or deteriorating, with some systems using 5 as a threshold and others using 3, the differences between the approaches are not very large [30]. Therefore, even slight miscalculations in MEWS scores can lead to disastrous or radically different outcomes. At present, although 100% accuracy can be obtained by GPT-4-Turbo after proper prompt engineering, clinician judgement should be considered in the triage process. With the help of clinicians, the calculated MEWS score can be rechecked, and higher specificity (84.8%, 95% CI 83.52%-86.1%) and sensitivity (72.4%, 95% CI 62.5%-82.7%) for the combined system have been suggested in patients with MEWS scores ≥4 [19]. Therefore, the primary objective of applying LLMs like ChatGPT in EDs is to assist nurses and physicians in more effectively and rigorously carrying out their duties and responsibilities. There is no need for health care professionals to feel pressured about being replaced by AI, as such a replacement is not feasible from both technological and ethical perspectives. The integration of LLMs is intended to enhance, not replace, the critical human elements of health care. For the GPT-4o model, the overall MEWS grading accuracy after prompt engineering improved to 96.20%, which can generally be considered acceptable. This is particularly notable when compared to the finding of a previous study, which indicated that the overall accuracy rate of nurses’ manual calculations was 81.8% [20]. Apart from this, during the experiment, we found that even when the previous conversation history was compiled and sent to ChatGPT again in a new chat, the chatbot failed to consistently follow the previous restrictive instructions. For instance, ChatGPT might not adhere to the previously provided MEWS grading criteria in subsequent chats. These issues can be resolved by including the restrictive prompt in every query, ensuring that the model consistently applies the correct guidelines. Future studies may evaluate the causes of this phenomenon and alert users to consider this issue when seeking more accurate responses.

Furthermore, we observed that when ChatGPT is provided with information from multiple patients concurrently, the output is automatically formatted as a table, where each patient is associated with a corresponding MEWS score. This structured output not only enhances the clarity of the results but also indicates the underlying potential for further integration into systems that require standardized data inputs, such as electronic health records and clinical dashboards. Future studies may examine the impact of proper prompt engineering efforts on further enhancing and standardizing the structured outputs.

### Findings of Outpatient Department Selection Guidance

In a previous report, out of a total of 2000 patients who visited EDs, the majority had relatively low MEWS scores (median score of 1) [31]. Therefore, outpatient department selection guidance, which can further direct patients in EDs with less urgent health conditions to the regular outpatient sector, is crucial for relieving pressure on EDs and helping allocate the limited ED resources to patients who truly need immediate care. In this part of the experiment, the GPT-4o model was chosen based on several advantages found during preliminary trials. First, ChatGPT’s responses based on the GPT-4o model exhibited richer and more anthropomorphic emotional expressions. For instance, when presented with the same patient scenarios, the GPT-4o model tended to provide more detailed explanations of several potential departments, discussing the reasons behind each option before making a recommendation, when compared to GPT-4-Turbo. According to a previous study, clinicians place greater value on a computer program’s ability to explain its reasoning than on its consistent diagnostic accuracy [32,33]. Moreover, in a few responses, the GPT-4o model expressed concerns for the patient, with phrases like “Wish the patient a speedy recovery,” which was rarely seen in the GPT-4-Turbo model. A high level of personification is greatly valued when selecting LLMs, as the real users in this application scenario are human patients seeking medical treatments. It has been shown that patient trust in health care providers is significantly related to their interpersonal communication skills [34]. This is a major reason for GPT-4o to be preferred over GPT-4-Turbo. Second, the response time of GPT-4o was suggested to be significantly shorter than that of GPT-4-Turbo [35]. Given the importance of time in EDs, a shorter response time translates to greater efficiency, which is crucial in emergency medical situations. Finally, according to OpenAI, GPT-4o–based ChatGPT has demonstrated better performance in handling languages other than English [16]. This multilingual capability makes the GPT-4o model a better choice for Chinese EDs, where the primary language is Chinese. Taking all these factors into account, only the GPT-4o model was used in the department triage guidance test. The overall performance of GPT-4o–based ChatGPT in providing outpatient department selection guidance was considered acceptable. The results showed that the overall accuracy rates for internal medicine and other specialized departments were the highest at around 93%. However, slightly lower accuracy (91.46%, 95% CI 86.50%-96.43%) was observed for general surgery departments. There are several potential reasons for this phenomenon. Many diseases in internal medicine and other specialized departments have more clearly defined and specific symptoms, such as respiratory infections and digestive problems. The presentation of these symptoms is usually more standardized, making it easier to correlate them with specific medical departments. For example, a cold is typically accompanied by symptoms, such as cough, runny nose, and fever, which are straightforward and easy to describe and recognize.

Symptoms related to surgical departments can be more complex and varied, with some overlapping with internal medicine problems (eg, abdominal pain can indicate a digestive issue in internal medicine or appendicitis requiring surgical intervention). To make a definitive decision in selecting general surgery departments, additional tests, such as imaging and laboratory tests, may be required. However, few patients entering the hospital have undergone these kinds of tests initially. Furthermore, there may be some internal reasons contributing to the differences in accuracy rates between departments. One key factor that limits the performance of the guidance is that the entire interaction is based solely on plain text. Although ChatGPT has the capability to analyze images, real-time communication with the chatbot is still unavailable. Visual inspection plays a crucial role in clinical diagnosis, including providing initial departmental triage. This limitation hinders the ability to fully assess and diagnose conditions that rely heavily on visual cues and real-time interaction. Additionally, insufficient training data on general surgery departments may also contribute to this discrepancy in accuracy. The lack of comprehensive and high-quality data can affect the model’s ability to accurately guide and diagnose surgical cases compared to other more thoroughly represented departments.

Another key finding in this part of the experiment was that the guidance provided by ChatGPT (GPT-4o model) was sometimes influenced by the medical history of patients, where physicians attributed 43.42% of the overall guidance error to an overreliance on the medical history of patients. For instance, a patient with a previous diagnosis of carcinoma of the cardia presenting to the hospital with symptoms of drowsiness and presleep hallucinations was guided by the chatbot to the Department of Gastrointestinal Surgery. This indicates that the chatbot might place too much emphasis on medical history over current symptoms, leading to potentially inappropriate department selection guidance. While medical history is crucial for the chatbot’s reference, it can occasionally introduce biases or inaccuracies in the guidance if not interpreted correctly in the context of current symptoms. In terms of specific department guidance, the highest accuracy (100%, across all 3 runs) was observed for the Department of Ophthalmology. This result is reasonable, as most diseases in the dataset in this department are straightforward and have little overlap with other departments. However, the Department of Dermatology had the lowest average accuracy rate at only 79.57% (95% CI 67.32%-91.82%). The diagnosis of dermatological diseases typically requires a combination of the patient’s symptoms and vital signs, such as the color, shape, and size of a rash. While a doctor can visualize this information, ChatGPT sometimes fails to correctly provide accurate guidance based solely on descriptions. Text-based input limits the chatbot’s ability to accurately interpret and guide diagnoses of conditions that heavily depend on visual inspection. In addition, while ChatGPT generally adhered to the historical instructions provided in our prompts, we observed occasional deviations that require further attention. Specifically, in some instances, the model recommended multiple departments simultaneously, despite our prompt explicitly stating that only 1 department should be recommended. Moreover, there were cases in which ChatGPT suggested a department outside of the predefined list. These “forgetting” issues highlight the challenges of ensuring strict adherence to specific instructions when using LLMs. We employed several system-level strategies to mitigate the issue of ChatGPT forgetting prior instructions. Specifically, all interactions were conducted as unique, standalone sessions, thereby ensuring that GPT-4o did not process multiple patient scenarios in a single session. Furthermore, for every request where GPT-4o provided a department selection, the preceding system instruction enforcing the 1-department constraint was included. Despite these measures, instances of the model recommending multiple departments still occurred. These problems underscore the need for further refinement in prompt engineering and model tuning to enhance the consistency and reliability of the system in clinical applications.

In practice, the AI-based triage system could function through 2 primary pathways. The first pathway involves patient triage kiosks and patient-facing interfaces. Upon arrival at the ED, patients could interact directly with an AI-driven kiosk interface. The kiosk would prompt the patient or their accompanying person to input essential physiological parameters, either manually or via connected medical devices (eg, automated blood pressure cuffs, pulse oximeters, and thermometers). The chatbot interface (eg, GPT-4o) would then instantly calculate the MEWS score. If the score is lower than the standard score, the chatbot will provide an initial triage recommendation along with appropriate departmental guidance. If the score is higher than the standard score, it will trigger a warning system to alert physicians and nurses for immediate action. This could help reduce waiting times and efficiently direct patients, particularly those with noncritical conditions, to relevant outpatient departments. The second pathway involves a streaming setup with human-in-the-loop validation. Alternatively or concurrently, the AI triage system could be deployed in a real-time streaming setup where nurses or clinicians supervise the recommendations generated by the chatbot. Patient information would be inputted by medical staff into an electronic health record system. The AI-generated triage recommendations would be presented to medical staff for rapid validation and final confirmation. Medical professionals could quickly approve, override, or adjust AI recommendations based on clinical judgment, ensuring patient safety and increasing clinical efficiency.

### Limitations

There were some limitations. First, we only included scenarios involving Chinese patients, and the outpatient department categories were based on Chinese regulations. Second, only 33 of the most common departments were included in this experiment, and some special departments were not included. Third, the patient scenarios provided to ChatGPT were sourced from a professional medical record database (Chinese Medical Case Repository), resulting in uniform and more structured descriptions. Additionally, in the time-pressured ED environment, the time-cost balance of the practical application of LLMs to assist triage needs to be further verified, along with consideration of the difference between real-world patient presentations, which may lack some patient information, and structured electronic health records.

### Future Studies

Future studies should focus on the performance of ChatGPT in other health care systems. Further expanding the research scope of the departments will improve the universality of the research. Further studies could focus on descriptions directly from patients, which are likely to be more varied and less structured. The ambiguity of real-world patient descriptions should be considered for practical usage of AI. Finally, while our observations suggest that GPT-4o tends to provide more human-like emotional responses, the impact of such emotional responses on user satisfaction, as well as the potential effects of system- and user-level prompt manipulations and parameter tuning, should be empirically and comprehensively evaluated.

### Conclusion

This study demonstrates that GPT-4–based ChatGPT models have significant potential for supporting patient triage and outpatient department selection in EDs in Chinese hospitals. Specifically, GPT-4-Turbo achieved an impressive accuracy of 100% after prompt engineering compared to GPT-4o’s overall accuracy of 96.2%, suggesting that GPT-4-Turbo may be more adaptable and responsive to targeted optimization. Although GPT-4o showed superior baseline responsiveness, its lower flexibility in optimization limited its peak performance relative to GPT-4-Turbo. Additionally, GPT-4o demonstrated acceptable accuracy in guiding outpatient department selection, with the highest accuracy in internal medicine departments and the lowest in general surgery departments. These findings highlight the crucial role of precise prompt engineering for clinical applications and support in integrating advanced LLMs into clinical workflows. However, further studies are warranted to evaluate their performance comprehensively in diverse health care settings and real-world conditions.

## Appendix

Multimedia Appendix 1 Sample patient triage analysis prompts used in this study.

Multimedia Appendix 2 Details of scores and differences for GPT-4o and GPT-4-Turbo.

Multimedia Appendix 3 General department guidance accuracy.

Multimedia Appendix 4 High resolution version of Figure 3. Kendall correlation coefficients between different runs for (A) GPT-4o and (B) GPT-4-Turbo models. Each cell represents the Kendall correlation coefficient between 2 specific runs, with values ranging from 0 to 1, where 1 indicates perfect positive correlation. The color intensity and size of the pie charts within each cell correspond to the strength of the correlation, with darker and fuller circles indicating higher correlation.

## Abbreviations

AI: artificial intelligence
AVPU: alert, response to voice, response to pain, unresponsive
ED: emergency department
ICU: intensive care unit
LLM: large language model
MEWS: Modified Early Warning Score

## Data Availability

The datasets generated during or analyzed during this study are not publicly available due to institutional case privacy and the presence of a large number of interaction dialogues but are available from the corresponding author upon reasonable request. Other than the sample prompts provided in Multimedia Appendix 1, additional detailed dialogues with ChatGPT may be obtained by contacting the corresponding author.
