# Supplementary material for: Patient Triage and Guidance in Emergency Departments Using Large Language Models: Multimetric Study
Source: J Med Internet Res. 2025 May 15;27:e71613. doi: 10.2196/71613 (PMC12123234; doi:10.2196/71613)
Supplement: Multimedia Appendix 2 [file jmir_v27i1e71613_app2.pdf]

Multimedia Appendix 2. Details of scores and differences for GPT-4o and GPT-4-Turbo.

Table S1. Accuracy of GPT-4o and GPT-4-Turbo on MEWS-score based patient triage accuracy before and after prompt engineering.

		Before prompt engineering		After prompt engineering	
		GPT-4-Turbo	GPT-4o	GPT-4-Turbo	GPT-4o
Accuracy	Run 1	50.54%	75.73%	100%	100%
	Run 2	96.60%	76.05%	100%	100%
	Run 3	57.44%	50.27%	100%	100%
	Run 4	94.55%	50.43%	100%	87.32%
	Run 5	48.81%	75.89%	100%	100%
	Run 6	75.13%	75.94%	100%	100%
	Run 7	48.60%	50.49%	100%	87.32%
	Run 8	26.59%	76.05%	100%	87.32%
	Run 9	45.63%	27.18%	100%	100%
	Run 10	95.36%	76.05%	100%	100%
	Average	63.93%	63.41%	100%	96.20%
Manual Grade Accuracy			81.80%		

Table S2. Average value of difference value of GPT-4o and GPT-4-Turbo on MEWS-score based patient triage accuracy before and after prompt engineering between GPT score and standard score.

		Before prompt engineering		After prompt engineering	
		GPT-4-Turbo	GPT-4o	GPT-4-Turbo	GPT-4o
Average Difference	Run 1	0.50	0.25	0	0
	Run 2	0.04	0.24	0	0
	Run 3	0.57	0.73	0	0
	Run 4	0.06	0.73	0	0.25
	Run 5	0.54	0.25	0	0
	Run 6	0.48	0.70	0	0
	Run 7	0.52	0.73	0	0.25
	Run 8	1.11	0.24	0	0.25
	Run 9	0.59	1.33	0	0
	Run 10	0.05	0.24	0	0
	Average	0.45	0.54	0	0.08
