# Supplementary material for: Patient Triage and Guidance in Emergency Departments Using Large Language Models: Multimetric Study
Source: J Med Internet Res. 2025 May 15;27:e71613. doi: 10.2196/71613 (PMC12123234; doi:10.2196/71613)
Supplement: Multimedia Appendix 3 [file jmir_v27i1e71613_app3.docx]

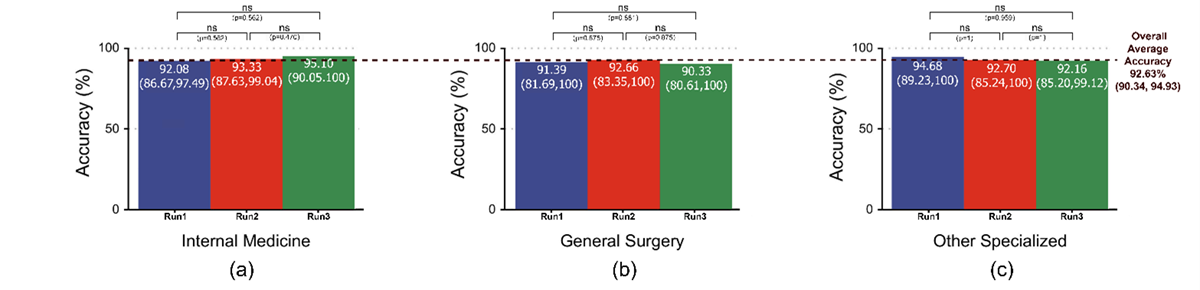


**Multimedia Appendix 3.** General department guidance accuracy. This figure presents the accuracy rates across three major hospital department categories. The vertical axis represents accuracy in percentage (%). Each bar within the subplots (a), (b), and (c) corresponds to accuracy values for individual runs (Run1, Run2, Run3) within each department category, respectively. Comparisons between runs within each department category are conducted using the Wilcoxon test, with p-values shown above the bars to indicate statistical significance (ns for non-significant results). The black dashed line represents the overall average accuracy across all departments.
