# Supplementary figures and images for: Patient Triage and Guidance in Emergency Departments Using Large Language Models: Multimetric Study
Source: J Med Internet Res. 2025 May 15;27:e71613. doi: 10.2196/71613 (PMC12123234; doi:10.2196/71613)

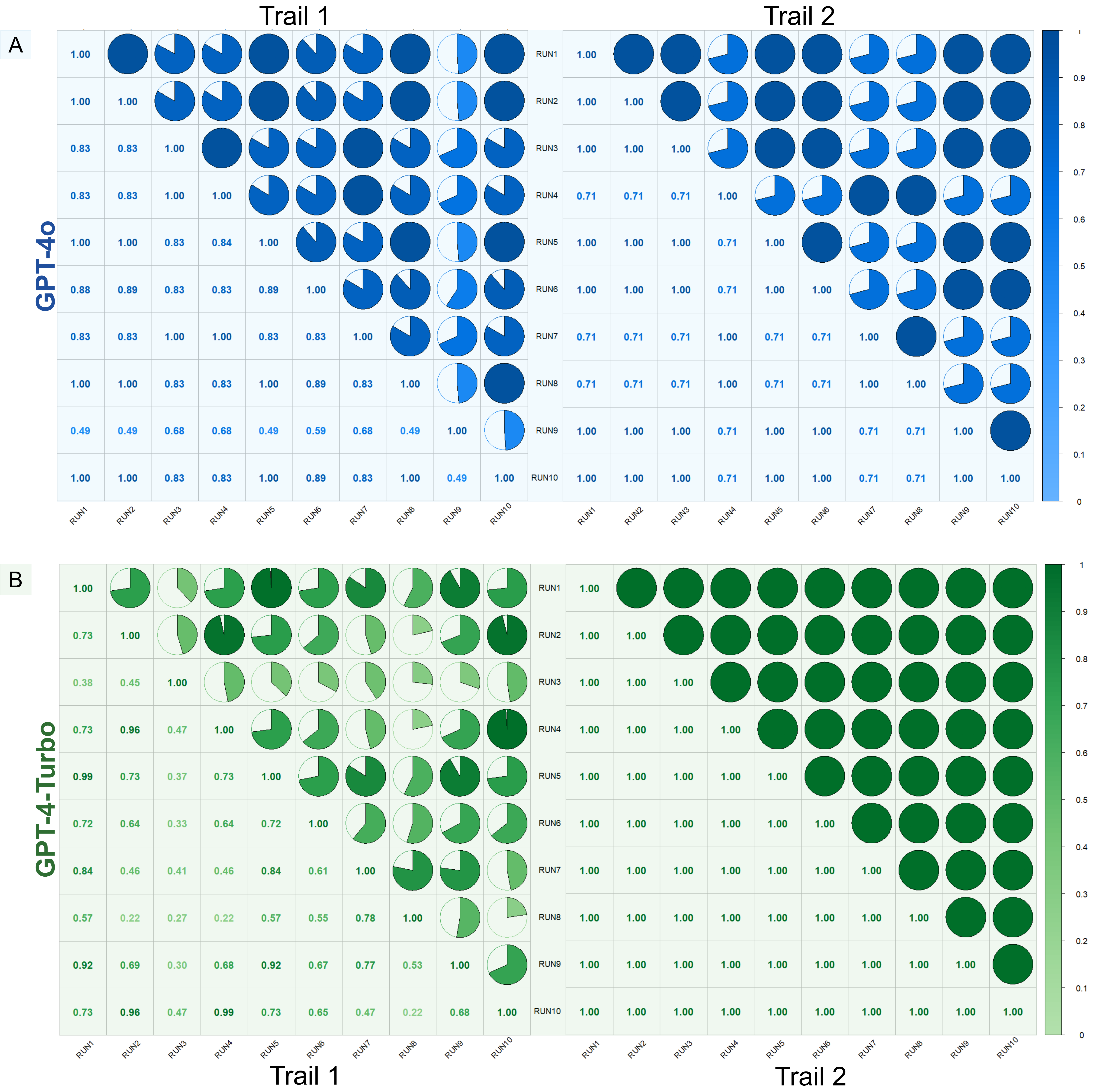

Supplement: Multimedia Appendix 4 [file jmir_v27i1e71613_app4.png]
